# Supplementary figures and images for: Antibiotic use and prescription and its effects on Enterobacteriaceae in the gut in children with mild respiratory infections in Ho Chi Minh City, Vietnam. A prospective observational outpatient study
Source: PLoS One. 2020 Nov 4;15(11):e0241760. doi: 10.1371/journal.pone.0241760 (PMC7641406; doi:10.1371/journal.pone.0241760)

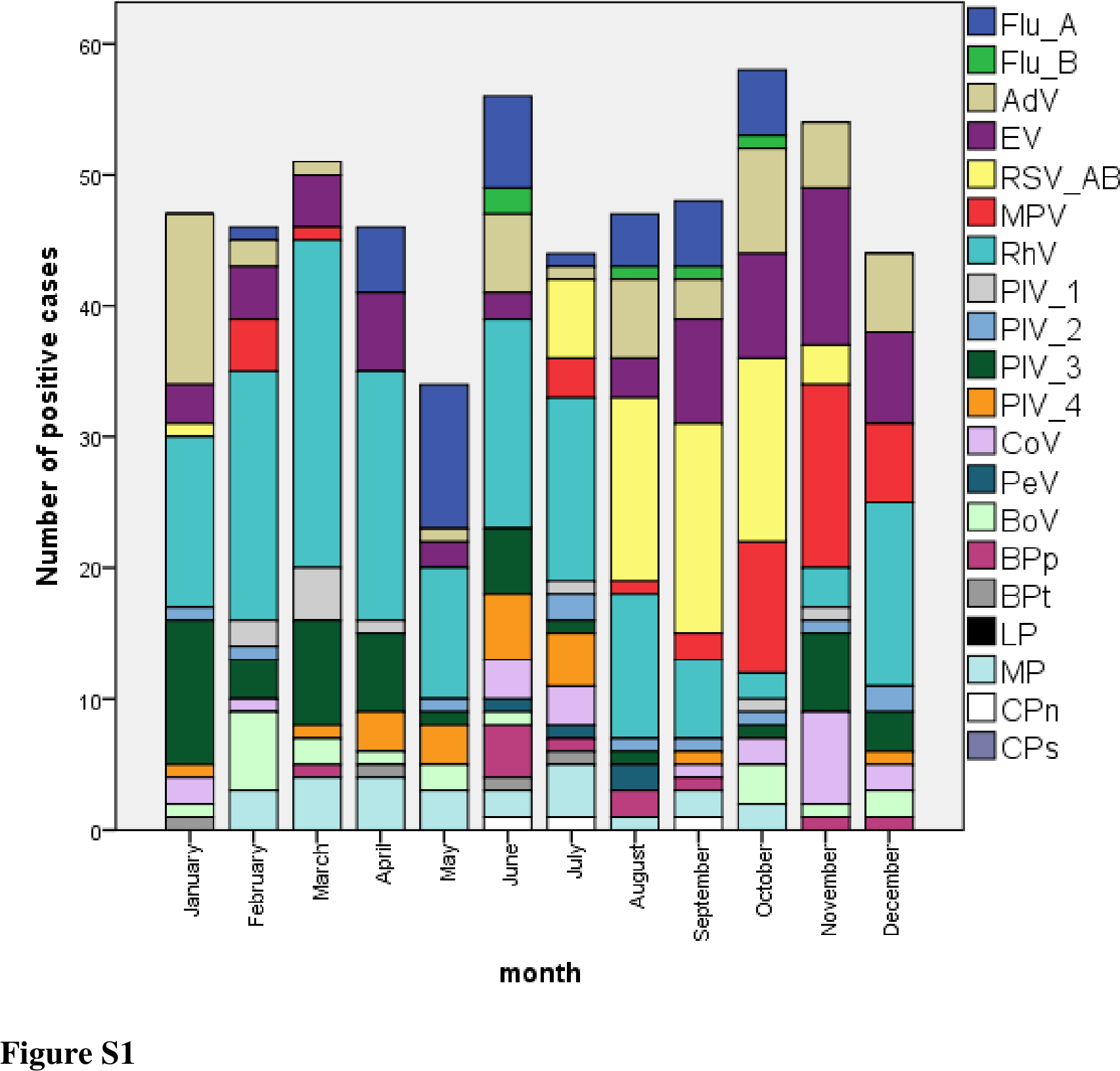

Supplement: S1 Fig — Detections by month of viral and atypical bacterial pathogens detected by real-time multiplex or single (RT-)PCR in pooled nasal and pharyngeal swabs taken at enrolment among 563 patients with acute respiratory infection enrolled at the outpatient department of Children’s Hospital 1, Ho Chi Minh City, Vietnam. X-axis: month; Y-axis: number of positive cases per pathogen. FluA: Influenza virus A; FluB: Influenza virus B; RSV A/B: Respiratory Syncytial Virus A and B; PIV1-4: Human parainfluenza viruses 1–4; hRV: Human Rhinovirus; EV: Enterovirus A-D; CoV: Human Coronavirus; BoV: Human Bocavirus; MPV: Human Metapneumovirus; PeV: Human Parechovirus; AdV: Adenovirus; MP: Mycoplasma pneumoniae; CPn: Chlamydophila pneumoniae; CPs: Chlamydophila psitacci; LP: Legionella pneumophila; BPt: Bordetella pertussis; BPp: Bordetella parapertussis. (TIF) [file pone.0241760.s001.tif]
