# Supplementary material for: Antibiotic use and prescription and its effects on Enterobacteriaceae in the gut in children with mild respiratory infections in Ho Chi Minh City, Vietnam. A prospective observational outpatient study
Source: PLoS One. 2020 Nov 4;15(11):e0241760. doi: 10.1371/journal.pone.0241760 (PMC7641406; doi:10.1371/journal.pone.0241760)
Supplement: Attachment — Submitted filename: Appeal.docx [file pone.0241760.s004.docx]

Dear Sir/Madam,

We would like to thank the editor Mehreen Arshad and one anonymous reviewer for their time to assess our manuscript.

In the reviews we were unable to establish the exact reasons for immediate rejection without giving us the opportunity to address any comments to improve the manuscript.

We have always understood that PLoS One is an inclusive journal, describing itself as a journal that believes "all rigorous science needs to be published and discoverable, widely disseminated and freely accessible to all".

The anonymous reviewer mentioned on two occasions that methods or statistics were not appropriate without giving an explanation or alternative. We have assumed this was the reason for answering “no” to the questions whether our manuscript was technically sound and whether statistics were appropriate. We have addressed these comments below and by adding information to the manuscript.

We think our science meets the criteria for rigorousness, even though the editor doesn’t think it adds new information to the research field.

We would therefore like to take this opportunity to appeal, and have included a point by point reply to the comments and addressing the issues raised.

We have indicated the editor’s and reviewer’s comments in bold and our replies to them by “>”

Judging from the comments, we suspect that many of the detected shortcomings were caused by previous rigorous cutting down of the number of words when submitting to another journal. We have now also taken the opportunity to bring back many of the information we had to cut out to comply with word limits.

We think the manuscript and described work will meet the criteria for publication in PLoS One.

Sincerely,

Prof. H. Rogier van Doorn
University of Oxford

Director of Oxford University Clinical Research Unit, Hanoi

PONE-D-19-28321
Antibiotic use and prescription and its effects on intestinal flora in children with mild respiratory infections in Ho Chi Minh City, Vietnam. A prospective observational outpatient study.
PLOS ONE

Dear Dr. van Doorn,

Thank you for submitting your manuscript to PLOS ONE. After careful consideration, we have decided that your manuscript does not meet our criteria for publication and must therefore be rejected.

I am sorry that we cannot be more positive on this occasion, but hope that you appreciate the reasons for this decision.

Yours sincerely,

Mehreen Arshad, M.D.
Academic Editor
PLOS ONE

Additional Editor Comments (if provided):

**The authors of this manuscript attempt to determine the overuse of antibiotics in children with URI. While the premise of this is interesting this study does not add new information to this research field.**

>We strongly believe that the systematic and comprehensive way in which we assessed several aspects of outpatient treatment of mild respiratory infections and the impact of antibiotic use adds to the field. While the high rates of antibiotic prescription for ARI in Vietnam are not new, assessing of pre-presentation antibiotic use and the comparison between reported and measured use (using HPLC in urine) certainly is in this setting. Furthermore, the semi-quantitative assessment of selection of resistant flora from longitudinal samples showing the negative impact of unnecessary antibiotic use using standard culture methods has to our knowledge not been done before and shows a very consistent and highly significant difference between resistant fractions among samples on presentation and after antibiotic use among >500 children against 7 out of 8 tested drugs, that was restored in a subset of patients after 28 days.

**The objective of this study is also unclear.**

>We have more explicitly clarified and expanded further on the objectives that were listed in abstract (line 5-9) and introduction (line 52-58)

**They have looked at resistant organisms very superficially.**

>We have expanded on the methods (lines 122-137, 147-149), interpretation and analysis (lines 233-254 and legends Figure 2 and table 4) to make clearer what was done and that this was not superficial. Semi-quantitative culture was used to assess the proportions of resistant colonies of lactose fermenting *Enterobacteriaceae* to 8 different drugs by culturing rectal swabs on MacConkey agar with and without antibiotics. More than 1100 faecal specimens were cultured and counted on 9 plates each.

**The 28 day follow up is only done on 35 kids without an explanation on why such few children were enrolled and how they were chosen.**

>This additional sample was collected from 35 of the last 40 patients who were enrolled into the study. This amendment to the protocol was made after a very strong selective effect was seen after interim analysis of the first 100 patients. Added to the manuscript in lines 70-73, 237-240)

**The authors have made several comments without showing any supporting data.**

>The editor did not indicate the instances where we made comments without supporting data. We have gone through the manuscript and think we have addressed these. There are now two instances left where we say “data not shown” or “unpublished data”. One concerns a discussion section of relative loads of *S. pneumoniae* and *H. influenzae* in samples from our study patients vs healthy volunteers form an unpublished study. We do not think including these data will contribute to the discussion or the justification of why these data weren’t used in the analysis of our study. The second concerns the exact data of switching regimens during different visits to the outpatient clinic. Although we don’t show the interpretation and analysis of these data, they are included in the raw dataset we uploaded.

**No details are given on how the molecular diagnostics were done.**

>References for the assays were given, we have published the use of these assays previously. We have added information from these previous publications and expanded on the described methods (line 92-110).

**Specific comments are below:

Introduction
Line 38: data from UK seems out of place, would recommend using Asian countries or those of similar economic status**

>Added examples from Vietnam in lines 41-51

**Methods:
Line 62: how was the follow up subset decided upon? Was there a selection criterion of some sort?**

>See above

**Line 66: was every patient enrolled in that 1 hr? Were they randomized in any way?**

>No randomisation was done. One study doctor was present at the outpatient clinic during this hour and enrolled the first eligible patient from the waiting list, helped the clinic doctors with consent and CRF completion and then moved on to enrol the next eligible patient etc.

**Line 89: Was the testing done as standard of care? Is this a commercial assay?**

>No and no, added to the manuscript (lines 92-93, 109-110)

**Results:
Line 126: This should be in a flow chart**

>Added as Figure 1

**Line 127-129: This sentence should either be deleted or the data shown.**

>Data were added (lines 161-164)

**Line 146-148: It is hard to believe that almost all children were colonized with S. pneumoniae, which then brings in to question the validity of the entire assay. No control data is shown either.**

>We have not used these data for analysis and have added more specific comments / justification / explanation on this in the discussion (lines 325-344).

**Line 168: It is unclear why the urine HPLC was done. It would have made more sense if the authors stated the objectives clearly in the beginning.**

>This is now stated more clearly in abstract (line 5-9) and introduction (line 52-58)

**The discussion is too wordy, and comes across as a policy paper in some places.**

>We have reviewed the discussion. We do not think it is too wordy for a journal without word limit. We think the policy parts (assuming the editor means lines 358-373) contain relevant context and contextual recommendations for the readers

**Figure 1: the x and y axis does not make sense. What is this a log of? CFU/ml? What does MC stand for?**

> MC stands for MacConkey agar, *i.e.* agar without antibiotics added, logs are of colony counts. This was stated in the legends. We have tried to show this clearer in both legends to figure 2 and methods (lines 135-137).

**There is no discussion of the supplementary figure or table in the main text.**

>Lines referring to this table and figure were moved / added to make their purpose and what is shown more clear (lines 55-58 and 189-194)


[Note: HTML markup is below. Please do not edit.]

Reviewers' comments:

Reviewer's Responses to Questions

**Comments to the Author**

1. Is the manuscript technically sound, and do the data support the conclusions?

The manuscript must describe a technically sound piece of scientific research with data that supports the conclusions. Experiments must have been conducted rigorously, with appropriate controls, replication, and sample sizes. The conclusions must be drawn appropriately based on the data presented.

Reviewer #1: No

2. Has the statistical analysis been performed appropriately and rigorously?

Reviewer #1: No

3. Have the authors made all data underlying the findings in their manuscript fully available?

The [PLOS Data policy](https://mail.oucru.org/owa/redir.aspx?C=gsSFSjePzm7gcXq8XtHEkczFuJWs_pHqZALUnu6HLOIg8DG9cZ3XCA..&URL=http%3a%2f%2fwww.plosone.org%2fstatic%2fpolicies.action%23sharing) requires authors to make all data underlying the findings described in their manuscript fully available without restriction, with rare exception (please refer to the Data Availability Statement in the manuscript PDF file). The data should be provided as part of the manuscript or its supporting information, or deposited to a public repository. For example, in addition to summary statistics, the data points behind means, medians and variance measures should be available. If there are restrictions on publicly sharing data—e.g. participant privacy or use of data from a third party—those must be specified.

Reviewer #1: Yes

4. Is the manuscript presented in an intelligible fashion and written in standard English?

PLOS ONE does not copyedit accepted manuscripts, so the language in submitted articles must be clear, correct, and unambiguous. Any typographical or grammatical errors should be corrected at revision, so please note any specific errors here.

Reviewer #1: No

5. Review Comments to the Author

Please use the space provided to explain your answers to the questions above. You may also include additional comments for the author, including concerns about dual publication, research ethics, or publication ethics. (Please upload your review as an attachment if it exceeds 20,000 characters)

**Reviewer #1: The manuscript prepared by Quang Minh et al presented a report on unnecessary usage of antibiotics in the Vietnamese population. The author summarized data collection on 563 children and evaluated the appropriateness of antibiotic use on presentation and proportion of resistant enterobacteriaceae in the gut flora before, during and after antibiotic prescription.**

**My major critique of the manuscript is the presentation and analyses of data do not align with the objectives of the manuscript. All objectives should be clearly mentioned in the introduction section.**

>Added in abstract (line 5-9) and introduction (line 52-58)

**The table and figures are not explained well in the results section, which creates a problem for a reader to understand the objectives and actual summary of the results. I would suggest to rewrite the result and discussion section.**

>We acknowledge this wasn’t done very well and has been changed, see also replies to editors comments. All figures are better referenced in the body of text, legends have been expanded where needed and parts of legends have been copied in the text for better reading and easier understanding.

**Regression analysis is not a suitable analysis for showing effect of antibiotics on normal flora.**

>The reviewer is of the opinion that something was done incorrectly in their view, without explanation or alternative. Statistical analysis to show difference between proportions of counts of resistant vs all cultured colonies was done using Wilcoxon matched pairs signed-rank test (see lines 147-149, 240-243 and legends to table 4) and regression was only used for visualisation of a mean line (lines 135-137, 243-245, legends to figure 2). We have made this clearer. We have also consulted a second statistician who confirmed the suitability of these analyses both for visual and statistical purposes. They commented the following on the use of the Wilcoxon matched pairs signed-rank test: “The low power of non-parametric tests in general reinforces the strength of these highly significant results.”

**Authors has not discussed which colonies they picked and no details were given on bacterial identification.**

>Added. Colonies were not picked, but counted. Colonies were not IDd but counted as *Enterobacteriaceae* based on shape, texture and colour (lines 130-135)

**Data is not substantial to support the semi-quantitative quantification of normal flora.**

> The reviewer is of the opinion that something was done incorrectly in their view, without explanation or alternative.

We have added more information on methods, analysis and interpretation and believe that both the data and the consistent and significant differences between timepoints and drugs are sufficiently robust and substantial.

**Figure S1 is about the seasonality of pathogens , I am confused, there is no discussion about seasonality.**

>Apologies, this sentence was cut out without making corrections to the figure. Text was restored and figure is referenced (lines 189-194)

**It was discussed out of context in line # 151.**

>See above. Apologies.

**Among pathogens detected Strep. Pneumonia (98%), H.influenzae (12.3%) and rhinovirus (27%), somehow the discussion on rhinovirus was left out.**

>We discussed why we did not include the bacteria in the analysis (lines 178-181, 322-341). We did not discuss all viral families separately, but have assessed appropriateness of antibiotic use taking viral PCR results into consideration for all viruses + atypical bacteria (lines 202-207)

**Similarly, table 4 is hard to understand. The author should explain a little bit about the fraction of bacteria and antibiotics.**

>We have tried to further clarify this, see lines 122-137, legends to figure 2 and table 4)

**Add a sentence for a rationale of semi-quantitative detection and how well it represents normal intestinal flora?**

>Added, lines 137-140

**It was not mentioned whether antibiotics were added in media or it was measured through disk diffusion?**

>Antibiotics were added to the media. We described this more clearly (lines 122-137 and legends to figure 2 and table 4)

**Molecular diagnostics: the details of methods should be given, with the name of the platform, principle of test and details of kits used.**

>Added in lines 92-110

**Overall, this manuscript requires language editing as sentence structure is not correct at various places and grammatical mistakes should be corrected.**

>We thank the author for this comment and suggestion and have done our best to check and improve the manuscript for correct use of the English language

**various sentences donot make sense for example:
line 180-182 212-213, 221-222**

>we have changed these and other sentences and hope they make sense to the reviewer now

**Specific comments:
Abstract:
Objective: line# 3-5 needs to be rephrased**

>Done (lines 3-7)

**The word moleculary should be changed to molecular detection**

>Changed “molecularly” to “using molecular assays” (line 10)

**Line#38: The term GP stands for..?**

>Clarified. GP stands for General Practitioner (line 40-41)

**Line #131-139: data is not shown in any table,**

>Correct, we do not feel repeating these numbers in a table would add significantly to the manuscript

**Line# 161: worse outcome should be defined if it is hospital discharge then analyses should be changed accordingly.**

>Outcome was defined and recorded by the outpatient physicians (line 158-161). Unclear what the reviewer means with hospital discharge in the context of outpatient care. Hospital admission was a (rarely) recorded outcome (1.2%, line 161).

6. PLOS authors have the option to publish the peer review history of their article ([what does this mean?](https://mail.oucru.org/owa/redir.aspx?C=P6KQ23mbrgt1UmPUsQKw1kxIfFtnVXaSrpwdnEsD2Zgg8DG9cZ3XCA..&URL=https%3a%2f%2fjournals.plos.org%2fplosone%2fs%2feditorial-and-peer-review-process%23loc-peer-review-history)). If published, this will include your full peer review and any attached files.


If you choose “no”, your identity will remain anonymous but your review may still be made public.


**Do you want your identity to be public for this peer review?** For information about this choice, including consent withdrawal, please see our [Privacy Policy](https://mail.oucru.org/owa/redir.aspx?C=k5078pzK09_Hd-sOrRZUzPfAVRlMC__HzIOzbaECMMYg8DG9cZ3XCA..&URL=https%3a%2f%2fwww.plos.org%2fprivacy-policy).

Reviewer #1: No


[NOTE: If reviewer comments were submitted as an attachment file, they will be attached to this email and accessible via the submission site. Please log into your account, locate the manuscript record, and check for the action link "View Attachments". If this link does not appear, there are no attachment files to be viewed.]

- - - - -
For journal use only: PONEDEC3
