# Supplementary material for: Antibiotic use and prescription and its effects on Enterobacteriaceae in the gut in children with mild respiratory infections in Ho Chi Minh City, Vietnam. A prospective observational outpatient study
Source: PLoS One. 2020 Nov 4;15(11):e0241760. doi: 10.1371/journal.pone.0241760 (PMC7641406; doi:10.1371/journal.pone.0241760)
Supplement: Attachment — Submitted filename: Response to Reviewers.docx [file pone.0241760.s005.docx]

PONE-D-19-28321R1
Antibiotic use and prescription and its effects on intestinal flora in children with mild respiratory infections in Ho Chi Minh City, Vietnam. A prospective observational outpatient study.
PLOS ONE

Dear Dr. van Doorn,

Thank you for submitting your manuscript to PLOS ONE. In response to your request, your revised manuscript was sent to 2 additional peer reviewers and I have now received feedback from both of them. After careful consideration, we feel that it has merit but does not fully meet PLOS ONE’s publication criteria as it currently stands. Therefore, we invite you to submit a revised version of the manuscript that addresses the points raised during the review process.

Please address the additional comments and in addition, it seems that Table 3b is missing from the manuscript.

Please ensure that your decision is justified on PLOS ONE’s [publication criteria](https://mail.oucru.org/owa/redir.aspx?C=7oJZuEAXFAWf4r6-R2UD8N7xdl-VfZ4f5TuFkCZZ6pd_m8N1Bw3YCA..&URL=https%3a%2f%2fjournals.plos.org%2fplosone%2fs%2fcriteria-for-publication) and not, for example, on novelty or perceived impact.

==============================

Please submit your revised manuscript by Jul 20 2020 11:59PM. If you will need more time than this to complete your revisions, please reply to this message or contact the journal office at plosone@plos.org. When you're ready to submit your revision, log on to https://www.editorialmanager.com/pone/ and select the 'Submissions Needing Revision' folder to locate your manuscript file.

Please include the following items when submitting your revised manuscript:

- A rebuttal letter that responds to each point raised by the academic editor and reviewer(s). You should upload this letter as a separate file labeled 'Response to Reviewers'.
- A marked-up copy of your manuscript that highlights changes made to the original version. You should upload this as a separate file labeled 'Revised Manuscript with Track Changes '.
- An unmarked version of your revised paper without tracked changes. You should upload this as a separate file labeled 'Manuscript'.

If you would like to make changes to your financial disclosure, please include your updated statement in your cover letter. Guidelines for resubmitting your figure files are available below the reviewer comments at the end of this letter.

If applicable, we recommend that you deposit your laboratory protocols in protocols.io to enhance the reproducibility of your results. Protocols.io assigns your protocol its own identifier (DOI) so that it can be cited independently in the future. For instructions see: [http://journals.plos.org/plosone/s/submission-guidelines#loc-laboratory-protocols](https://mail.oucru.org/owa/redir.aspx?C=MBTgkFqhE3FF4XOe42T7Cms3id6dosjo0ENu_sF7S6B_m8N1Bw3YCA..&URL=http%3a%2f%2fjournals.plos.org%2fplosone%2fs%2fsubmission-guidelines%23loc-laboratory-protocols)

We look forward to receiving your revised manuscript.

Kind regards,

Jane Foster, PhD
Academic Editor
Point by point reply

1.) Please ensure that your manuscript meets PLOS ONE's style requirements, including those for file naming. The PLOS ONE style templates can be found at http://www.plosone.org/attachments/PLOSOne_formatting_sample_main_body.pdf and http://www.plosone.org/attachments/PLOSOne_formatting_sample_title_authors_affiliations.pdf

**>I have reformatted tables and figures and affiliations according to these requirements**

2.) We noted in your submission details that a portion of your manuscript may have been presented or published elsewhere. "Validation of the HPLC detection methods of antibiotics in urine was described in 10.1002/bmc.4699 (als referenced in manuscript). The total percentage of positive samples (aggregated and without clinical metadata an d questionnaire results) was mentioned in this manuscript" Please clarify whether this [conference proceeding or publication] was peer-reviewed and formally published. If this work was previously peer-reviewed and published, in the cover letter please provide the reason that this work does not constitute dual publication and should be included in the current manuscript.

**>We have added a paragraph on this in the cover letter:**

**Some data presented in this study have been published previously in a technical paper describing validation of the methods. This paper was peer reviewed and published in Biomedical Chromatography 34:e4699. This paper describes development of HPLC methods to detect antibiotics in urine and analysis of samples collected in our study. The paper deals almost entirely with technical and validation aspects of the methods and only has one paragraph and one figure (pasted below) where clinical evaluation and results on clinical samples are presented but only in an aggregated manner and without the context of clinical metadata and questionnaire results:**

**“The urine samples were collected from 563 pediatric patients under 16 years of age (50% of patients were less than 2 years old, and 95% of patients under 5 years old). The validated methods were successfully applied to determine the six β‐lactams in urine samples (10 patients with severe ARIs were anuria). Figure 2 presents the results of a qualitative measurement of the β‐lactams in clinical samples. Among of the tested β‐lactams, CFI was detected at the highest rate (54/553–9.8%), followed by amoxicillin (52/553–9.4%), while CFU was the least common identified medication, at only 1.8% (10/553).”**

**FIGURE 2	The determination of six β‐lactams in clinical urine samples. (a) Percentage of urine samples positive with at least one of six β‐lactam antibiotics tested. (a) negative; (b) positive with one antibiotic; (c) positive with more than one antibiotic (N = 553). (b) Frequency of β‐lactams that HPLC methods detected in urine samples of pediatric patients with ARIs.**

**We reference the methods and their validation in the methods section of our paper and we present the results in context of their clinical metadata and the questionnaire results and therefore we believe this does not constitute double publication.**

3.) In your Data Availability statement, you have not specified where the minimal data set underlying the results described in your manuscript can be found. PLOS defines a study's minimal data set as the underlying data used to reach the conclusions drawn in the manuscript and any additional data required to replicate the reported study findings in their entirety. All PLOS journals require that the minimal data set be made fully available. For more information about our data policy, please see http://journals.plos.org/plosone/s/data-availability.

Upon re-submitting your revised manuscript, please upload your study’s minimal underlying data set as either Supporting Information files or to a stable, public repository and include the relevant URLs, DOIs, or accession numbers within your revised cover letter. For a list of acceptable repositories, please see http://journals.plos.org/plosone/s/data-availability#loc-recommended-repositories. Any potentially identifying patient information must be fully anonymized.

Important: If there are ethical or legal restrictions to sharing your data publicly, please explain these restrictions in detail. Please see our guidelines for more information on what we consider unacceptable restrictions to publicly sharing data: http://journals.plos.org/plosone/s/data-availability#loc-unacceptable-data-access-restrictions. Note that it is not acceptable for the authors to be the sole named individuals responsible for ensuring data access.

We will update your Data Availability statement to reflect the information you provide in your cover letter.

**>We have now uploaded our dataset with the submission**

4.) Please amend your list of authors on the manuscript to ensure that each author is linked to an affiliation. Authors’ affiliations should reflect the institution where the work was done (if authors moved subsequently, you can also list the new affiliation stating “current affiliation:….” as necessary).

We note that you have included affiliation numbers 1 - 10 however affiliations 8 does not have an author linked to it. Please amend affiliation 8 to link an author to it or remove if added in error.

**>Thank you for spotting this, this has been corrected**

5.) We note that you have included the phrase “data not shown” in your manuscript. Unfortunately, this does not meet our data sharing requirements. PLOS does not permit references to inaccessible data. We require that authors provide all relevant data within the paper, Supporting Information files, or in an acceptable, public repository. Please add a citation to support this phrase or upload the data that corresponds with these findings to a stable repository (such as Figshare or Dryad) and provide and URLs, DOIs, or accession numbers that may be used to access these data. Or, if the data are not a core part of the research being presented in your study, we ask that you remove the phrase that refers to these data.

**>The two occasions where we mentioned “data not shown” have been corrected. In the first instance about antibiotic regimen change we deleted the sentence, in the second instance on Streptococci and Haemophilus we deleted “(data not shown)” as the full data set has been uploaded now.**

6.) Please include your tables as part of your main manuscript and remove the individual files. Please note that supplementary tables should be uploaded as separate "Supporting Information" files.

**>This has been corrected**

7.) PRTC Notes I'm not sure if those comments also need to be added or it has already been sent back to author before.

**>I have assumed there was nothing to address here**

8). Please provide additional details regarding participant consent. In the ethics statement in the Methods and online submission information, please ensure that you have specified (1) whether consent was informed and (2) what type you obtained (for instance, written or verbal). If your study included minors, state whether you obtained consent from parents or guardians. If the need for consent was waived by the ethics committee, please include this information.

Please also clarify whether your study was specifically reviewed and approved by your IRB.

**>This was all already included in the methods section, subsections “Patients and samples” and “Ethics approval”.**

2. Is the manuscript technically sound, and do the data support the conclusions?

The manuscript must describe a technically sound piece of scientific research with data that supports the conclusions. Experiments must have been conducted rigorously, with appropriate controls, replication, and sample sizes. The conclusions must be drawn appropriately based on the data presented.

Reviewer #2: Yes

Reviewer #3: Yes

3. Has the statistical analysis been performed appropriately and rigorously?

Reviewer #2: Yes

Reviewer #3: Yes

4. Have the authors made all data underlying the findings in their manuscript fully available?

The [PLOS Data policy](https://mail.oucru.org/owa/redir.aspx?C=9xbtDOAyKlsTrtz4JY8duHINiWcRGyDBfFlz1lGzsYl_m8N1Bw3YCA..&URL=http%3a%2f%2fwww.plosone.org%2fstatic%2fpolicies.action%23sharing) requires authors to make all data underlying the findings described in their manuscript fully available without restriction, with rare exception (please refer to the Data Availability Statement in the manuscript PDF file). The data should be provided as part of the manuscript or its supporting information, or deposited to a public repository. For example, in addition to summary statistics, the data points behind means, medians and variance measures should be available. If there are restrictions on publicly sharing data—e.g. participant privacy or use of data from a third party—those must be specified.

Reviewer #2: Yes

Reviewer #3: Yes

5. Is the manuscript presented in an intelligible fashion and written in standard English?

PLOS ONE does not copyedit accepted manuscripts, so the language in submitted articles must be clear, correct, and unambiguous. Any typographical or grammatical errors should be corrected at revision, so please note any specific errors here.

Reviewer #2: Yes

Reviewer #3: Yes

6. Review Comments to the Author

Please use the space provided to explain your answers to the questions above. You may also include additional comments for the author, including concerns about dual publication, research ethics, or publication ethics. (Please upload your review as an attachment if it exceeds 20,000 characters)

Reviewer #2: Antibiotic resistance is a global concern with one of the major contributors being the over-prescription of antibiotics. The current study shines a light on this issue by focusing on antibiotic use and prescription in children with mild respiratory infections in Ho Chi Minh City, Vietnam. To address this issue, the authors enrolled patients and recorded diagnoses, prescribed therapy and outcome at visit 1 and on follow-up after 7 days. Respiratory bacterial and viral pathogens were also detected using molecular assays. Antibiotic use before presentation was assessed using questionnaires and urine HPLC. The main finding reported is the overuse and over-prescription of antibiotics for uncomplicated respiratory infections.

This is an important topic and while the results are not surprising, they are still interesting and worth reporting. I have the following additional queries and recommendations:

(1) The extent to which these findings can be generalised to jurisdictions where antibiotics are available on prescription only needs to be considered more in the discussion. While the over-prescription aspect might be common, over the counter use varies quite a lot.

**>We have added a sentence to the Limitations section on this**

(2) I agree with the authors that an important part of this study is the measurement of antibiotics in urine. However, the sensitivity of the HPLC assay is very low at 49% and argues against the interpretation provided in the discussion that it may provide a more accurate and higher detection rate of antibiotic use than the questionnaire.

**>We have rephrased this sentence**

(3) Following on from this point, the authors also make a somewhat flawed leap in logic by stating that they can assume on the basis of this low sensitivity that antibiotic use is twice as high as detected in patients where no antibiotic use was reported. This needs to be removed from the discussion.

**>We think that with the rephrasing of the previous sentence, this assumption / interpretation can still be made albeit with caution**

(4) The use of the term ‘gut flora’ is problematic as this description has been superseded by ‘gut microbiota’. In any case, since they have only assessed specific members in a targeted way, the analysis falls short of the current gold standards for a global assessment of the microbiota. This makes the title a little misleading and I suggest removing the reference to the gut flora altogether as it will create expectations that are not met with the analysis.

**>We have removed this from the manuscript and instead refer to this work using “intestinal *Enterobacteriaceae*”**

Reviewer #3: The authors of this paper have nicely showed the misuse of antibiotics in uncomplicated ARI in Vietnamese children. They have also determined the presence of different common respiratory viral and bacterial pathogens; as well as went on to determine the presence of antibiotics in their urine. Lastly they have also established the association of “selection of antibiotic-resistant members of Enterobacteriaceae in gut” with antibiotic use. I think that their data will further highlight the irrational overuse of antibiotics in ARI and its effect on the resistance development and selection in microbiota.

I am suggesting some minor edits to further improve the reader’s experience of this paper.
1. Though mentioned in abstract and results but please also mention the sample collection period in methods section.

**>Sorry to disagree here, but I don’t think the dates of the actual delivery of methods should be in the methods, as it is a result in my opinion. Happy to add if the editor / reviewer insists, but I don’t think it belongs in methods.**

2. Line 165-176 and lines 200-213. Under both these subheadings, though you have nicely defined results in the text form but would be nice if authors can also present their data in form of tables or figures, either in the paper or in supplementary information.

**>We have now included all figures/numbers from these sections in the tables**

3. Lines 190-191. Please mention the whole virus name before using abbreviations. Similarly in the associated-figure also mention viral abbreviations.

**>This has been corrected**

4. Figure-2 was incorrectly marked as “Figure-1”.

**>Apologies, this has been corrected**
